# The Impact of Agreeableness Trait on Volunteer Service Motivation and Behavior: A Moderated Mediation Study of Chinese College Students

**DOI:** 10.3390/bs15101308

**Published:** 2025-09-25

**Authors:** Chen Chen, Weilin Su

**Affiliations:** School of Literature, Capital Normal University, Beijing 100089, China; 5917@cnu.edu.cn

**Keywords:** agreeableness trait, volunteer service behavior, volunteer service motivation, social support, moderated mediation, volunteering, motivation, personality traits

## Abstract

How to continuously motivate college students to participate in voluntary activities has always been one of the burning issues in the field of educational psychology research. Based on the activation theory, this study constructed and tested a moderated mediation model to explore how to improve college students’ volunteer service motivation and behavior from the perspective of agreeableness traits and examined the moderating role of social support. By collecting three-wave time lagged data from 408 Chinese college students (59.6% female, age 18–22, means = 19.8, SD = 1.2), SPSS and Amos software were used to test the research hypotheses and the whole model. The results show that agreeableness traits of college students have a significant positive influence on their volunteer service motivation and behaviors. Volunteer service motivation mediates the positive influence of agreeableness traits on volunteer service behaviors. The social support perceived by college students positively moderates the impact of agreeableness traits on their volunteer service motivation and then promotes their volunteer service behaviors. These findings not only enrich the research literature on college students’ personality traits, volunteer service, and social support, but also provide some suggestions on how to motivate college students to participate in volunteer service from the three aspects of personality, motivation, and social support.

## 1. Introduction

In recent years, with the in-depth promotion of innovative moral education at Chinese universities and the continuous support of volunteer services from all sectors of society, college students have become the backbone of the volunteer service field (Lv et al., 2024). According to the 2023 China Volunteer Service Development Index Report, college students account for 56.8% of registered volunteers nationwide, playing an important role in more than thirty service areas such as rural revitalization, community governance, and large-scale events (L. Chen et al., 2023). Taking the 2022 Beijing Winter Olympics as an example, college students accounted for 94% of the 18,000 volunteers (Jin & Tang, 2024). They not only played an irreplaceable role in the successful hosting of the event but also demonstrated the dedication and sense of responsibility of Chinese college students in the new era (L. Chen et al., 2023). In this context, studying the factors influencing college students’ participation in volunteer service will not only contribute to the continuous development of China’s youth volunteer cause (Zhou et al., 2024), but also provide empirical evidence for optimizing volunteer management strategies, which has important theoretical and practical value.

For college students, participating in volunteer services is not only a contribution to society, but also an important way for personal growth and development (Wondimu & Admas, 2024). On the one hand, by participating in volunteer activities, college students can personally feel their responsibilities and obligations as members of society, thereby stimulating their attention and thinking on social issues. The cultivation of this sense of responsibility will help them perform their duties more actively in their future careers (Choi et al., 2023). On the other hand, relevant research in the field of educational psychology further shows that participating in volunteer activities can meet students’ three major psychological needs of autonomy, competence, and belonging (Cruce & Moore, 2007), effectively relieve anxiety and depression, enhance psychological resilience, and transform it into an internal driving force for academic and career development (Marks & Jones, 2004). Therefore, focusing on the research of college students’ volunteer service can not only provide empirical support for optimizing volunteer management and promoting the development of youth volunteer work, but also has dual value for the personal growth and sustainable development of college students.

The functionalist theory of volunteering emphasizes that a volunteer activity can satisfy multiple motivations at the same time, and in order to maintain volunteer engagement, these activities should be consistent with the volunteer’s primary motivational function (Snyder et al., 1999). Additionally, the existing research implies that college students’ participation in volunteer activities is influenced by various factors, which can be categorized into individual traits and external environmental factors (Lee & Won, 2011). Agreeableness is a prosocial trait describing how one interacts with others (Carlo et al., 2005), and people with this trait tend to be more prosocial, compassionate, and willing to help others (Akhtar, 2019). Hence, they have stronger motivations to participate in volunteer activities and show more volunteer service behaviors. Moreover, the trait activation theory emphasizes that appropriate external environment can effectively activate an individual’s internal traits, thereby enhancing or weakening the influence of traits on behaviors (Tett & Burnett, 2003). Social support focuses on the “support and understanding for an individual’s behavior from family, friends and other significant people” (J. M. Chen et al., 2012). For students, if they can feel the social support for their participation in voluntary activities from their surroundings (Li et al., 2025), their motivation for volunteering will accordingly increase, thus leading to more active involvements in voluntary activities.

**Figure 1:**
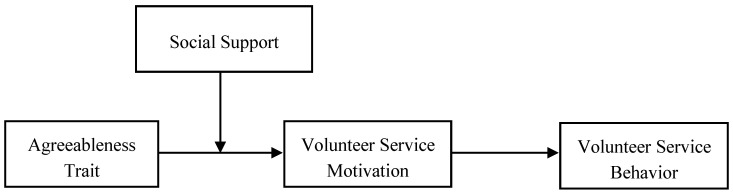
The proposed research model.

In conclusion, this study attempts to build and validate a moderated mediation effect model of agreeableness of college students on their volunteer service behaviors (see Figure 1) and also investigates the mediating role of volunteer service motivation and the moderating role of social support. On the one hand, this study expands the application of trait activation theory in volunteer service contexts and reveals how situational cues regulate the expression of traits; on the other hand, it conducts a cross-cultural verification of the relationship between agreeableness and volunteer service behavior in the unique institutional context of Chinese higher education.

## 2. Theoretical Framework and Research Hypotheses

### 2.1. Agreeableness Traits and Volunteer Service Behavior

Volunteer service behavior, an important manifestation of individuals practicing social values, refers to the prosocial actions that individuals take spontaneously based on their recognition of the public interest without any external coercion (C. W. Moore & Allen, 1996). It has dual attributes of value orientation and practical interactivity (Mak et al., 2022). Many existing studies have shown that there is a significant correlation between volunteer service participation and individual psychological traits (Ackermann, 2019; Harp et al., 2017), among which personality traits, as deep psychological structures, play a fundamental role in shaping behavioral motivations (Q. P. Chen et al., 2021). The agreeableness dimension under the Big Five personality theory framework, as a core trait reflecting an individual’s prosocial tendencies, includes four key elements: empathy, trust, prosocial motivation, and willingness to cooperate (Carlo et al., 2005; Stavrova et al., 2023). These elements together constitute the psychological mechanism that drives volunteer participation (Dionigi et al., 2020).

Specifically, college students with high agreeableness traits present a unique logic of action (Reizer et al., 2023). At the cognitive level, they have a higher sensitivity to the needs of others and can accurately identify the needs of service recipients through situational interpretation. At the emotional level, their empathy response threshold is lower, making it easier for them to resonate emotionally and stimulate a willingness to help others. At the behavioral tendency level, in a teaching situation that emphasizes cooperative learning, students with high agreeableness traits clearly prefer cooperative problem-solving rather than competitive strategies (Wilmot & Ones, 2022). In other words, the agreeableness personality trait group is highly consistent with the essential characteristics of volunteer activities—volunteer services require participants to have the empathy to understand the situation of the service recipients, to maintain prosocial motivation to continue to invest, and are more dependent on cooperation skills in teamwork (Cho et al., 2024). Therefore, the following research hypothesis is proposed:

**H1.**  *Agreeableness traits of college students have a significant positive influence on their volunteer service behavior.*

### 2.2. The Mediating Role of Volunteer Service Motivation

Volunteer service motivation, as the direct cause and the real driving force for individuals to participate in volunteer service activities (Anderson & Moore, 1978), may vary with time and the individuals under the influence of different value orientations or individual characteristics (Stunkel & Grady, 2011). Existing research has confirmed that personality traits with strong prosocial qualities (such as agreeableness) can make volunteers more likely to empathize (Windon et al., 2024), thus enhancing their volunteer service motivation. Meanwhile, college students with agreeableness traits generally form a personal philosophy and behavior standard of valuing justice and concerning others’ interests, which will drive them to proactively show cognitions and behavior tendencies beneficial both to themselves and others (Bang & Ross, 2009), thus providing internal energy for the formation and development of their volunteer service motivation. Therefore, the following research hypothesis is proposed:

**H2.**  *Agreeableness traits of college students have a significant positive impact on their volunteer service motivation.*

Among the diverse factors influencing individual volunteer service behaviors, volunteer service motivation is undoubtedly the core factor (Maki & Snyder, 2017). Scholars have proven that individual volunteer service motivation not only has a positive impact on volunteer service behavior, but also effectively conveys the impact of individual factors such as background on their volunteer service behavior (Cady et al., 2018). Concurrently, relevant research based on motivation theory has confirmed that an individual’s motivation usually depends on his or her personality traits and the work drive attracted or triggered by the task itself, which directly affects the individual’s attitude and behavior (Miao et al., 2025). In other words, volunteer service motivation, as the specific application of individual motivation in the field of volunteer service, will not only be stimulated by the students’ agreeableness, but can also encourage students to devote themselves to the voluntary activities and thus make corresponding voluntary behaviors. Therefore, the following research hypothesis is proposed:

**H3.**  *Volunteer service motivation mediates the relationship between agreeableness and volunteer service behavior of college students.*

### 2.3. The Moderating Role of Social Support

Social support perceived by college students participating in voluntary activities emphasizes “the level of support and understanding from their families, friends and other significant individuals for their participation in voluntary activities” (Rathakrishnan et al., 2022). Relevant research in the field of social support has confirmed that individuals who can perceive sufficient social support in their surroundings are more inclined to make prosocial behaviors, while when individuals feel socially ostracized or lack social support, their prosocial behaviors will significantly decrease (Trombini et al., 2025). This suggests that for college student volunteers, whether they can receive support from their families, friends, and other important figures for their participation in voluntary activities is very important (Cao et al., 2024). In addition, the trait activation theory emphasizes that external contextual factors influence the externalization process of individuals’ personality traits externalizing into self-motivation and behaviors (Tett et al., 2021). If individuals can obtain conditions consistent with their personality traits from the external environment, the process and effect of externalizing traits can be further enhanced (Manteli & Galanakis, 2022). Therefore, this study believes that the degree of support that college students receive from their families, friends, and other significant individuals for their participation in voluntary activities can have a significant impact on the relationship between agreeableness and their volunteer service motivation. Therefore, the following research hypothesis is proposed:

**H4.**  *The social support perceived by college students can moderate the positive influence of agreeableness on their volunteer service motivation.*

Combining the previous discussions about the mediating role of college students’ volunteer service motivation and the moderating role of social support, this paper believes that the social support perceived by college students further moderates the mediating role of volunteer service motivation in the relationship between agreeableness and their volunteer service behaviors. Specifically, when college students perceive a high level of support from their surroundings for their participation in volunteer service activities (De León & Fuertes, 2007), the stimulative effect of agreeableness on their volunteer service motivation is stronger, thus enhancing their volunteer service behaviors to a higher level. On the other hand, when college students perceive that the people around are not friendly regarding their participation in voluntary activities, due to the poor positive effect of agreeableness on their volunteer service motivation (MacNeela, 2008), less volunteer service behavior is transmitted through volunteer service motivation. Therefore, the following research hypothesis is proposed:

**H5.**  *The social support perceived by college students can moderate the mediating effect of volunteer service motivation in the relationship between agreeableness and their volunteer service behaviors.*

## 3. Methods

### 3.1. Procedure and Participants

This study was conducted in accordance with the principles of the Declaration of Helsinki and approved by the Ethics Committee of the School of Literature, Capital Normal University (Reference number: CNU20240308002; Approval date: March 2024). To verify the research hypotheses and theoretical framework, this study conducted online questionnaire surveys on 500 college students from a comprehensive university in Beijing, China, from January to April 2025. The sample was selected based on the university’s strategic position as an important provider of regional volunteer services—the average annual scale of its volunteer delivery in the past three years accounted for more than half of the total number of volunteers in the regional university system. Specifically, with the assistance of relevant personnel from the university’s student management agency, this study randomly selected college students to participate in the survey and obtained their contact information and email addresses. Each student then received a personalized email explaining the research process. At the same time, all participants provided digital informed consent, with explicit clarification of data anonymity and withdrawal rights.

In addition, to further reduce the impact of common method bias on the research results, this study collected sample data at three different time points, with a two-week interval between each time point (Conway & Lance, 2010). At time 1, college students were asked to complete the agreeableness traits questionnaire and provide their demographic information, including gender, age, and grade. At time 2, the participating college students were asked to fill out the questionnaire again, including their volunteer service motivation and the social support they felt. At time 3, participating college students were invited to take part in a follow-up assessment to measure their volunteer service behaviors. After removing invalid questionnaires with incomplete information, obvious response patterns, and unmatched responses in the surveys, a total of 408 valid questionnaires were retained, with the effective rate of 81.6%. In the final valid sample, there were 165 males (40.41%) and 243 females (59.6%), and the average age of the participating students was 18 to 22 years old (means = 19.8 years, standard deviation = 1.2). In terms of the grade distribution of the tested college students, the result was relatively average, with 100 freshmen (24.5%), 102 sophomores (25%), 112 juniors (27.5%), and 94 seniors (23%). Finally, to validate the adequacy of the final sample (N = 408) and assess result representativeness, the post hoc power analysis was conducted using GPower (Cohen, 2013). The analysis evaluated three effect sizes: large (*f*^2^ = 0.35), medium (*f*^2^ = 0.15), and small (*f*^2^ = 0.02). At a significance level of 0.05, the statistical power for the full regression model predicting volunteer behavior reached 0.88, exceeding the 0.8 threshold recommended in the prior literature (Cunningham & McCrum-Gardner, 2007; Su & Zhang, 2023). This confirms that the sample size provides sufficient sensitivity to detect even small effects, supporting the robustness and generalizability of the study’s conclusions.

### 3.2. Measures

The main variables involved in this study include agreeableness traits, social support, and volunteer service motivation and behavior, all of which are measured using the maturity scale. All measurement tools were translated in both directions to ensure cross-cultural equivalence, and the adaptability of the measurement items was verified by item information curve analysis.

#### 3.2.1. Agreeableness Traits

The 3-item agreeableness trait scale comes from the brief version of the big five personality scale (NEO-FFI) compiled by Costa and McCrae (1992), which includes “Kind-hearted, know how to be grateful”, “Willing to help, kind to people”, and “Trust others, straightforward and honest”. In this study, a five-point Likert scale is used, ranging from strongly disagree (1) to strongly agree (5). The agreeable trait of college students is measured by calculating the average score. The higher the average score, the more obvious the agreeable personality trait. In the current study, the Cronbach’s alpha for scores from agreeableness traits was 0.765.

#### 3.2.2. Volunteer Service Motivation

The 30-item volunteer service motivation scale was compiled by Clary et al. (1998), which includes the 6 dimensions of values expression, knowledge understanding, social interaction, career, self-protection, and self-enhancement. In this study, a five-point Likert scale is used, ranging from strongly disagree (1) to strongly agree (5). The average score of these items was calculated to measure students’ volunteer service motivation. The higher the score, the stronger the motivation for volunteer service of the surveyed college students. In the current study, the Cronbach’s alpha for scores from volunteer service motivation was 0.790.

#### 3.2.3. Volunteer Service Behavior

The 4-item volunteer service behavior scale was compiled by Carlo et al. (2005), with typical items including “I often participate in voluntary activities”. In this study, the students who participated in the survey were asked to choose the descriptions provided based on their own actual situation using a five-point Likert scale ranging from very inconsistent (1) to very consistent (5). The higher the score, the more volunteer service behaviors the interviewed college students exhibited. In the current study, the Cronbach’s alpha for scores from volunteer service behavior was 0.887.

#### 3.2.4. Social Support

The 12-item social support scale adopted is compiled by Canty-Mitchell and Zimet (2000), which includes the three dimensions, support from family, friends, and other significant individuals. This paper adopts the Likert five-point rating method, ranging from very inconsistent (1) to very consistent (5), and calculates the average score of these items to measure the social support perceived by students. The higher the score, the more support the interviewed college students feel for participating in volunteer activities. In the current study, the Cronbach’s alpha for scores from social support was 0.828.

### 3.3. Data Analysis

All statistical analyses of this study were conducted using SPSS 24.0 and Amos 24.0 software. To be specific, we first conducted a confirmatory factor analysis (CFA) via Amos 24.0 to test the discriminant validity of the theoretical model. It usually uses the ratio of Chi-Square/degree of freedom (χ^2^/*df*, 2.000 or less), the comparative fit index (CFI, 0.900 or more), the goodness of fit index (GFI, 0.900 or more), Tucker–Lewis index (TLI, 0.900 or more), normative fit index (NFI, 0.900, or more), the root means square error of approximation (RMSEA, 0.080 or less), and standardized root mean square residua (SRMR, 0.080 or less) (C. Chen et al., 2022; L. T. Hu & Bentler, 1999). Meanwhile, the average variance extracted (AVE) and composite reliability (CR) were also introduced to further evaluate the reliability and validity of this study (Bagozzi & Yi, 1988). Then, Harman’s single-factor test was used to check for common method bias of the current study (Kock, 2020). Furthermore, the descriptive statistics and correlation analyses were used to preliminary test the relationships between agreeableness traits, social support, volunteer service motivation, and volunteer service behavior. Finally, the multiple linear regression was used to examine the relationship between the variables. In addition, this study further validated the entire moderated mediation model using the bootstrap method using the PROCESS program and Model 7 developed by Hayes (2015).

## 4. Results

### 4.1. Confirmatory Factor Analyses

The current study firstly conducts confirmatory factor analyses to test the validity of the proposed model. The results are presented in Table 1. It can be observed that the four-factor measurement model (with agreeableness, volunteer service motivation, volunteer service behavior, and social support as four independent factors) exhibits a better fit to the data (χ^2^/*df* = 1.622 < 2.000, GFI = 0.953 > 0.900, CFI = 0.976 > 0.900, NFI = 0.941 > 0.900, TLI = 0.971 > 0.900, RMSEA = 0.039 < 0.080, and SRMR = 0.058 < 0.080) than the other three measurement models, which demonstrates that the respondents have a good discriminant validity in the current study. Furthermore, the average variance extracted (AVE) and composite reliability (CR) metrics for all study variables met the recommended thresholds (AVE > 0.5, CR > 0.7), confirming the convergent validity and reliability of the measured constructs, as evidenced by prior research.

### 4.2. Common Method Bias Analysis

To test the common method bias, Harman’s single-factor test was used to examine the data obtained from the survey, and all items were put together to carry out the factor analysis. The result showed that one factor extracted and explained only for 32.04% of the variance, which was less than the suggested criterion of 50%. Therefore, the sample data obtained in this study does not have the problem of excessive interpretation of a single factor. Besides, the results of confirmatory factor analysis combining all the indexes also exhibit a poor fit to the data (χ^2^/*df* = 13.047, RMSEA = 0.172, GFI = 0.637, TFI = 0.439, CFI = 0.524, TLI = 0.439, and SRMR = 0.199), with fitting index well below evaluating index. Hence, the common method bias is not a major issue and the measurement scales used in the current study have good reliability and validity.

### 4.3. Descriptive Statistics and Correlation Analysis

Table 2 shows the means, standard deviations, and intercorrelations for the main variables. As predicted in this study, agreeableness is positively correlated not only with college students’ volunteer service motivation (r = 0.383, *p* < 0.01), but also volunteer service behavior (r = 0.163, *p* < 0.01). Besides, college students’ volunteer service motivation is also significantly and positively related with their volunteer service behavior (r = 0.277, *p* < 0.01). Moreover, social support they perceived is also significantly and positively related to their volunteer service motivation (r = 0.419, *p* < 0.01) and behavior (r = 0.277, *p* < 0.01). Taken together, these results can provide preliminary evidence for the relationship among the main variables and foundation for the subsequent regression analysis and model testing in the current study.

### 4.4. Hypotheses and Model Testing

Table 3 shows the results of regression analysis of each variable. It should be noted that before calculating the interaction terms, the mean centering method was used to alleviate the potential multicollinearity problem (Aiken et al., 1991). Variance inflation factor (VIF) analysis further confirmed the absence of multicollinearity, as the VIF scores of all predictors in the regression model were less than 3.0 (range: 1.102–1.876). As for the direct impact of agreeableness on volunteer motivation and behavior, it can be seen from Model 2 and Model 5 in Table 2 that, on the basis of control variables, agreeableness has a significantly positive influence on volunteer service motivation (β = 0.387, *p* < 0.001) and volunteer service behavior (β = 0.143, *p* < 0.01). Therefore, Hypothesis 1 and Hypothesis 2 are supported.

Furthermore, for the mediating effect of volunteer service motivation, this study followed Baron and Kenny’s procedures to verify the mediating effect (Baron & Kenny, 1986). On the basis of Model 4 and Model 5, volunteer service motivation was put into the regression equation and Model 6 was obtained. The result shows that volunteer service motivation of college students has a significant positive effect on their volunteer service behavior (β = 0.263, *p* < 0.001) and agreeableness has a non-significant positive effect on volunteer service behavior (β = 0.042, *p* > 0.05). These indicate that volunteer service motivation completely mediates the positive influence of agreeableness on volunteer service behavior. Therefore, Hypothesis 3 is supported.

**Figure 2:**
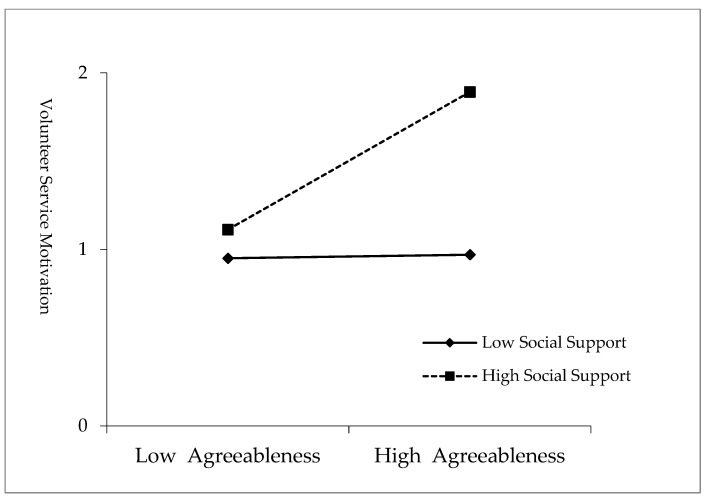
Moderating effect of social support in the relationship between agreeableness and volunteer service motivation.

Furthermore, after the variables’ decentration of agreeableness and social support, the interaction term was constructed. Then, on the basis of Model 2, the interaction term was put into the regression equation and Model 3 was obtained. The result shows that the interaction term has a significant positive effect on volunteer service motivation (β = 0.190, *p* < 0.001), indicating that social support plays a significant positive moderating role in the influence of agreeableness on volunteer service motivation. To further demonstrate the moderating effect, Figure 2 shows the moderating effect of social support in the relationship between agreeableness and volunteer service motivation with one standard deviation positive and negative. As can be seen from the graph, the higher the social support college students can perceive, the more significantly can agreeableness improve volunteer service motivation. While the degree of social support they perceive is weak, the influence of agreeableness on volunteer service motivation is faint. Therefore, Hypothesis 4 was supported.

Finally, this study applied bootstrap methods and learned from scholars Hayes and Preacher (Hayes, 2015; Hayes & Preacher, 2010) to verify the moderated mediating model. To be specific, this study used Model 7 in the PROCESS program, 5000 samples, and 95% confidence intervals for bootstrapping. The results are presented in Table 4. The result shows that the whole model test index with volunteer service motivation as the mediating role and social support as the moderating role is 0.0521, 95% CI = [0.0121, 0.1035], excluding zero. This suggests that social support college students perceive moderates the mediating role of volunteer service motivation between agreeableness and volunteer service behavior. Specifically, when the mean value of social support is subtracted by one standard deviation, 95% CI of the overall moderated mediating effect is [−0.0243, 0.0633], containing zero, while when the mean value of social support is increased by one standard deviation, 95% CI of the overall moderated mediating effect is [0.0502, 0.1926], excluding zero. It can be inferred that the mediating effect of volunteer service motivation in the relationship between agreeableness and volunteer service behavior becomes more significant with the increase of social support college students perceive. Taken together, Hypothesis 5 is well supported.

## 5. Discussion

This study adopted a three-stage stratified sampling method to conduct an empirical test on 408 Chinese college students. By constructing a moderated mediation effect model, it systematically verified the mechanism of the effect of agreeableness traits on volunteer service motivation and behavior of college students. The results confirmed that agreeableness traits significantly and positively predicted volunteer service motivation and behavior. Volunteer service motivation could mediate the positive impact of agreeable personality on volunteer service behavior. Social support not only positively moderated the stimulation intensity of agreeableness traits on volunteer service motivation, but also significantly strengthened the mediating effect of motivation variables in the personality–behavior transmission path. These findings deepen the theoretical understanding of the driving mechanisms of volunteer service behavior and provide empirical evidence for promoting volunteer service practice.

### 5.1. Theoretical Implications

This study makes several theoretical contributions to the literature on personality traits, volunteer activity participation, and perceived support. Firstly, college students with agreeableness have higher volunteer service motivation and will be more proactive in volunteer service behavior. As a prosocial personality trait, agreeableness can influence college students’ thinking styles and value judgments to a certain extent (Carlo et al., 2005; Reizer et al., 2023). Generally, this kind of college students are often more enthusiastic, sympathetic, cooperative, helpful, and more sensitive to the needs of others (Paiman et al., 2024), which is more likely to form cognition and behavior tendencies that benefit others. Furthermore, they display stronger volunteer service motivation and are thus more actively involved in voluntary activities, accordingly demonstrating more volunteer service behavior.

Secondly, the volunteer service motivation of college students fully mediates the positive impact of agreeableness on their volunteer service behavior. In other words, the influence of agreeableness on the volunteer service behavior of college students is exerted by stimulating their volunteer service motivation, which is consistent with the previous research (L. Huang et al., 2025) and further verifies that volunteer service motivation is a key factor affecting volunteer service behavior (Lee, 2012). Specifically, college students with high agreeableness traits produce a stronger motivation to participate in voluntary activities. Furthermore, in order to meet their own service motivation needs, those with stronger volunteer service motivation usually continue to participate in volunteer service activities and demonstrate more volunteer service behavior (Costello et al., 2017).

Finally, the social support perceived by college students can moderate the impact of agreeableness on their volunteer service motivation and behavior. Specifically, the higher the degree of support from family, friends, and other significant individuals for their participation in voluntary activities perceived by college students, the more motivated college students with agreeableness will be to participate in voluntary activities and exhibit more volunteer service behaviors (Kramer et al., 2021). Few existing research has discussed the moderation role of social support perceived by college students and its effect on the relationship between personality traits and volunteer service behaviors from the perspective of trait activation theory (e.g., Palmer et al., 2017; Smillie et al., 2006; Zou et al., 2025). This study enriches the research perspectives of volunteer service behavior and provides theoretical support for universities to further protect the volunteer service rights and interests of college students and create a volunteer service atmosphere (Teoh & Hilmert, 2018). In addition, it should be pointed out that social support may have both positive and negative effects on volunteering behavior. Moderate support (such as peer encouragement) enhances intrinsic motivation by satisfying autonomy, while excessive support (such as overprotective family involvement) may trigger “support overload” and reduce the willingness to participate autonomously. This is consistent with the “too much is too little” effect proposed by Pierce and Aguinis (2013), whereby support exceeding the optimal level may be counterproductive, which also contributes to the research results on the impact of social support.

### 5.2. Managerial Implications

As important bridges and platforms for college students to participate in social practice and voluntary activities, universities have an irreplaceable influence on the voluntary behavior of college students (E. Moore et al., 2014). Based on the results of this study, it is recommended that colleges and universities should start with the following three aspects to further enhance the initiative and participation of college students in volunteer services.

Firstly, given the positive impact of agreeableness trait on volunteer service, colleges and universities can design service projects that better suit the personality traits of students with high agreeableness traits. For example, they can organize volunteer services that require a high degree of empathy, such as accompanying lonely elderly people and caring for children with special needs, to fully tap the emotional advantages of individuals with high agreeableness traits. At the same time, it is also important to continuously optimize the incentive mechanism for volunteer services, taking into account both material and emotional aspects (Ouyang et al., 2024). For example, by sending thank-you letters, service certificates, etc., the need for interpersonal affirmation of those with high agreeableness can be satisfied. This kind of emotional satisfaction is often more motivating than material rewards for students with high agreeableness traits. In addition, the exemplary role of those with high agreeableness traits should be brought into play. Through their mentoring and guidance, we can fully play the role of role models and leaders, thereby creating a campus atmosphere where everyone participates in volunteer services and forms a virtuous cycle of volunteer service ecology.

Secondly, according to the conventions of educational intervention research (Hattie, 2008) an effect size β ≥ 0.200 is considered substantive. In this study, both the agreeableness → motivation (β = 0.387, *p* < 0.001) and motivation → behavior (β = 0.263, *p* < 0.001) pathways met this standard, indicating that enhancing agreeableness traits to stimulate motivation and, in turn, promote volunteering behavior is feasible. While the direct effect of agreeableness → behavior (β = 0.143, *p* < 0.001) was statistically significant, it required the mediation effect of motivation to have a substantive impact, suggesting that relying solely on the cultivation of agreeableness traits is insufficient to maximize volunteering participation. Hence, after realizing volunteer service motivation is an important internal factor that propels college students to participate in volunteer service activities (Leisink et al., 2021), the universities should help college students fully understand the functions and roles of volunteer service, guide students to build prosocial-oriented volunteer service motivation, and transform their incorrect cognition and self-serving motivation from the students’ perspective by various means such as indirect ideological guidance and moral education. Meanwhile, college students should not only improve their own moral cultivation and social responsibility awareness, establishing an altruism-oriented volunteer service motivation, but also personally participate in volunteer service activities or public welfare activities (Perry et al., 2008), experiencing the spiritual satisfaction brought by helping others and further strengthening their willingness for long-time participation. This two-pronged approach, combining institutional support with experiential learning, strengthens students’ long-term commitment by integrating personal growth with societal contribution, fostering a sustainable culture of volunteerism rooted in mutual benefit.

Finally, in respect of social support, universities should provide college student volunteers with sufficient material, rights, and interests guarantee, as well as necessary care and positive feedback both spiritually and materially (Su et al., 2022). Besides, it is beneficial to help students sift through reliable, credible voluntary channels, encourage students to participate in volunteer service activities as a unit of families, dormitories, and others, and harness the power of role models around by propagating their voluntary experience through approaches like new media, lectures, and campus showcases to encourage college students to learn from the advanced deeds and hence create a philanthropic voluntary atmosphere at campus and in society (Abuiyada, 2018), which further motivates students to participate in voluntary activities. In addition, it is also important to note that the main task of college students is still studying, which means that the school’s support for college students’ participation in volunteer activities should be moderate. Students should be encouraged to make full use of their spare time and actively participate in volunteer activities on the premise of completing their studies.

### 5.3. Limitations and Recommendations

Despite the theoretical and managerial contributions described above, this study has several limitations. Firstly, the sample of this study only covers college students from one university in China, which raises concerns about its cultural representativeness. It is worth noting that traditional Chinese culture has shaped a unique psychological structure (J. Huang, 2024), which is reflected in the localized expression of agreeableness (e.g., harmony-oriented conflict resolution) and differentiated volunteer cognition (e.g., obligatory participation) (L. Chen et al., 2023). While these cultural specificities enhance contextual relevance, they simultaneously constrain the model’s cross-cultural applicability. Crucially, the study does not propose culturally embedded mechanisms to explain how agreeableness or social support operate distinctively within China’s volunteer ecosystem—such as the role of *guanxi* networks in amplifying social support effects. Consequently, generalizations beyond similar cultural contexts require caution. Future research should retest this theoretical model using multinational samples to assess its general validity or develop culture-specific pathways to explain the observed patterns. Secondly, although this study theoretically constructed an integrated model of the relationship between agreeableness traits and volunteer service and adopted a multi-stage data collection method to improve the reliability of the research conclusions, all data were obtained through self-assessment by college students, making it impossible to truly infer the causal relationship between the variables. Furthermore, the measurement scales used in this study were relatively simplified. For example, agreeableness was measured with only three items, while volunteering behavior was measured with four items. This may not adequately capture these constructs and the complexity of these variables. Therefore, this study suggests that future research should adopt mixed measurement methods (combining self-reports with other evaluations), longitudinal or experimental designs, to further accurately grasp the causal relationships between core variables. Finally, this study only explored the influencing mechanism of college students’ volunteer service from the perspective of agreeableness traits. However, the factors that affect college students’ participation in volunteer activities may be extensive and diverse, for example environmental factors (J. Hu et al., 2023) and individual factors (Aguila & Salvador, 2025). Therefore, future research can explore the influencing factors and mechanisms of college students’ participation in volunteer activities in a more in-depth and comprehensive manner from the perspective of the interaction between the environment and the individual.

## 6. Conclusions

This study used a cross-time point design to explore the impact of agreeableness trait on volunteer service behavior of Chinese college students and constructed and verified a moderated mediation model to discuss in detail the moderating role of social support and the mediating role of volunteer service motivation in this influence process. The main conclusions are as follows: (1) agreeableness traits have a significant positive influence on volunteer service motivation and behaviors; (2) volunteer service motivation mediates the positive influence of agreeableness traits on volunteer service behaviors; (3) social support positively moderates the impact of agreeableness traits on volunteer service motivation and then promote volunteer service behaviors. Future research should further explore the mechanisms by prioritizing longitudinal designs to establish causality, explore multigroup SEM for cross-cultural validation, and test hybrid interventions balancing extrinsic rewards with altruistic growth. These findings have important theoretical and practical implications for offering a sustainable framework for ethical volunteerism rooted in both individual development and societal benefit.

## Figures and Tables

**Table 1:** Results of confirmatory factor analyses.

Model	χ^2^	*df*	χ^2^/*df*	RMSEA	NFI	GFI	CFI	TLI	SRMR
Four-factor model	158.98	98	1.622	0.039	0.941	0.953	0.976	0.971	0.058
Three-factor model	456.29	101	4.518	0.093	0.831	0.845	0.862	0.836	0.093
Two-factor model	611.94	103	5.941	0.110	0.773	0.804	0.802	0.770	0.110
Single-factor model	1356.91	104	13.047	0.172	0.497	0.637	0.524	0.439	0.199

**Table 2:** Descriptive statistics and correlation coefficients of variables.

Variables	Means	SD	Agreeableness	Social Support	Service Motivation	Service Behavior
Agreeableness	2.51	0.884	1			
Social Support	2.62	0.906	0.464 **	1		
Service Motivation	2.41	0.848	0.383 **	0.419 **	1	
Service Behavior	3.21	1.088	0.163 **	0.279 **	0.277 **	1

Note: N = 408; ** *p* < 0.01.

**Table 3:** Results of multiple regression analyses.

Variables	Volunteer Service Motivation			Volunteer Service Behavior		
	Model 1	Model 2	Model 3	Model 4	Model 5	Model 6
Gender	0.128 **	0.045	0.069	0.068	0.037	0.025
Grade	−0.048	−0.100 *	−0.081	0.105 *	0.086	0.113 *
Agreeableness		0.387 ***	0.200 ***		0.143 **	0.042
Volunteer Service Motivation						0.263 ***
Social Support			0.270 ***			
Agreeableness × Social Support			0.190 ***			
R^2^	0.019	0.159	0.267	0.016	0.035	0.093
ΔR^2^		0.140	0.108		0.019	0.058
F	3.884 *	25.442 ***	29.300 ***	3.227 *	4.871 **	10.343 ***

Note: N = 408; * *p* < 0.05, ** *p* < 0.01, *** *p* < 0.001.

**Table 4:** Results of bootstrap analyses.

Dependent Variable	Moderating Indirect Effect					Moderating Mediating Effect			
	Moderator	Index	SE	95%		Index	SE	95%	
				LLCI	ULCI			LLCI	ULCI
Volunteer Service Behavior	Low Social Support	0.0177	0.0220	−0.0243	0.0633	0.0518	0.0236	0.0121	0.1035
	High Social Support	0.1116	0.0361	0.0502	0.1926				

Note: N = 408; bootstrap sample size = 5000. LL = lower limit; CI = confidence interval; UL = upper limit.

## Data Availability

Data supporting the reported results are available from the authors on request.
